# Phenanthroline-Based Reversible Fluorescent Probe for Ultrasensitive and Selective Detection of Ni^2+^ and Mitochondrial Imaging

**DOI:** 10.3390/molecules31111815

**Published:** 2026-05-25

**Authors:** Jing Huang, Xinyan Yu, He Zhao, Fenying Kong, Yong Dai

**Affiliations:** School of Chemistry and Chemical Engineering, Yancheng Institute of Technology, Yancheng 224007, China; 15295338498@163.com (J.H.); yxy494903874@outlook.com (X.Y.)

**Keywords:** Biphen, nickel (II), mitochondria, ultratrace

## Abstract

In this study, bis-1,10-phenanthroline (Biphen) was synthesized via a hydrogen transfer-mediated coupling reaction in a single step. The resulting compound was demonstrated, for the first time, to function as a selective fluorescent probe for Ni^2+^ ions. The presence of Ni^2+^ at a 2:1 molar ratio of Biphen to Ni^2+^ results in complete fluorescence quenching, with a detection limit of 4.34 × 10^−9^ M in aqueous medium. Fluorescence is restored upon the introduction of a suitable chelating agent, producing an “on-off-on” fluorescence switching response. Furthermore, fluorescence co-localization studies demonstrate that Biphen functions as a mitochondria-targeted fluorescent probe with excellent cell membrane permeability, enabling rapid, reversible imaging and ultratrace detection of Ni^2+^ in mitochondria of live cells. Overall, this work demonstrates highly selective ultratrace detection of Ni^2+^ in both aqueous and biological environments, providing a promising platform for mitochondrial imaging and potential diagnostic applications.

## 1. Introduction

Nickel is an important element owing to its widespread applications in biological systems, industrial processes, and catalytic reactions [1,2,3,4]. In biological systems, nickel plays an essential role in various metalloenzymes and is involved in protein structure and function, particularly in prokaryotic organisms [5,6]. However, excessive exposure and bioaccumulation of nickel can lead to severe toxicological effects, including skin allergies, lung fibrosis, acute pneumonitis, and neoplastic transformations, as well as damage to the kidneys and cardiovascular system [7,8,9]. Moreover, nickel is present at trace levels in various food sources, including raw meat, chocolate, hydrogenated oils, milk and dairy products, and canned foods [10]. Although the molecular mechanisms underlying nickel-induced carcinogenesis remain incompletely understood, studies by the Goetzman group suggest that nickel exposure may contribute to mitochondrial damage [11]. Mitochondria are membrane-bound organelles essential to all eukaryotic cells and play a critical role in numerous physiological and pathological processes [12]. Their functions are closely associated with key cellular events, including cell differentiation, aging, and apoptosis, and mitochondrial dysfunction has been implicated in a wide range of human diseases, including diabetes, cancer, and neurodegenerative diseases [13]. Consequently, the development of efficient and selective methods for detecting nickel ions is of considerable importance for environmental monitoring and protecting human health.

The development of target-specific chemosensors has attracted considerable interest in both chemistry and biochemistry owing to their ability to provide rapid, nondestructive detection of metal cations and enable in situ real-time analysis through fluorescence enhancement or quenching. Although numerous fluorescent chemosensors for detecting transition and heavy metal ions have been developed over the past decade [14,15,16,17,18,19,20,21], relatively few studies have focused on the selective detection of Ni^2+^ ions in aqueous media [4,10,19,22,23,24,25,26,27,28,29]. These studies have established a foundational framework for Ni^2+^ recognition system design; however, the currently reported Ni^2+^ fluorescent probes still exhibit several inherent limitations that restrict their practical and biological applicability. In many cases, probe fabrication involves multi-step and complex synthetic routes, resulting in increased preparation costs and limited scalability. Additionally, numerous probes display relatively high detection limits, thereby failing to achieve ultratrace-level sensitivity. A significant proportion also lacks reversible sensing capability and adequate selectivity, often exhibiting cross-reactivity toward competing metal ions. Furthermore, only a limited number of probes possess mitochondrial targeting functionality, which critically constrains the investigation of nickel-induced mitochondrial dysfunction at the subcellular level. Therefore, there remains an urgent need to develop advanced Ni^2+^ fluorescent probes that integrate synthetic simplicity, ultratrace detection sensitivity, high selectivity, reversible response, and mitochondrial targeting capability, thereby enabling practical applications in environmental monitoring and biological systems.

N-Heteroarene-based fluorescent probes have attracted widespread attention owing to their excellent optical properties, ease of synthesis, and ability to exhibit distinct colorimetric and fluorescence changes in the presence of specific metal ions [16,19,30,31,32,33,34]. Among various N-Heteroarenes, 1,10-phenanthroline (Phen) exhibits several distinctive features, including aromaticity, rigidity, planarity, basicity and strong chelating ability, which have led to its widespread application in catalysis, coordination chemistry, and supramolecular chemistry [35,36]. In addition, the phenanthroline scaffold, which integrates both signal transduction and recognition sites, represents an attractive platform for the development of intensity-based optical probes. Its rigid structure also provides an entropic advantage in metal chelation compared with the more flexible 2,2′-bipyridine ligand. Consequently, numerous phenanthroline derivatives have been employed for the selective detection of metal ions [37,38,39,40,41,42]. However, their application as highly selective, ultratrace, and mitochondria-targeted fluorescent probes for Ni^2+^ remains largely unexplored.

**Scheme 1:**
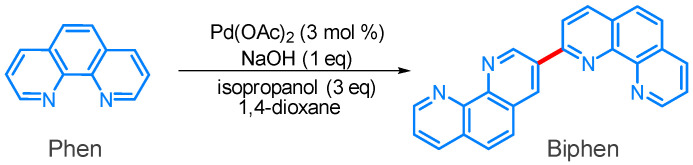
Palladium-catalyzed hydrogen transfer-mediated synthesis of Biphen.

As part of our ongoing efforts toward the development of structurally unique N-heterocycles and chemical sensors [16,43,44,45,46,47,48], we synthesized 2,3′-bi(1,10-phenanthroline) (Biphen) via a hydrogen transfer-mediated coupling reaction under palladium-catalyzed conditions in a single step [43] (Scheme 1). Compared with monomeric phenanthroline-based probes, this dimeric scaffold incorporates two phenanthroline moieties, thereby enhancing structural rigidity and molecular stability. Its extended π-conjugated system can suppress non-radiative energy dissipation and improve fluorescence performance, while its distinctive spatial configuration confers favorable molecular recognition capability. These advantageous photophysical and structural properties indicate that Biphen has considerable potential as a fluorescent platform for metal ion detection. We report, for the first time, the development of Biphen as a high-performance fluorescent probe for the selective and ultratrace detection of Ni^2+^. This probe exhibits an ultralow detection limit of 4.34 × 10^−9^ M in a CH_3_OH–H_2_O system, high selectivity toward Ni^2+^ over competing metal ions and a rapid, reversible “on-off-on” fluorescence switching response. It exhibits markedly improved selectivity and sensitivity compared with previously reported fluorescent probes based on monomeric phenanthroline frameworks [37]. Importantly, Biphen demonstrates inherent mitochondrial targeting, low cytotoxicity, and excellent photostability, enabling real-time and reversible imaging of Ni^2+^ in the mitochondria of living cells, an ability rarely achieved by existing Ni^2+^ probes. This work extends the application scope of phenanthroline dimers for metal ion fluorescent sensing and introduces an effective platform for Ni^2+^ detection in both environmental and biological systems. Biphen enables selective ultratrace detection of Ni^2+^, exhibiting a pronounced fluorescent quenching response with sensitivity down to the ppb level in aqueous media. Furthermore, it supports fluorescence imaging and real-time monitoring of Ni^2+^ in living cells using an inverted fluorescence microscope. These findings highlight the strong potential of Biphen for Ni^2+^ analysis in complex biological environments and provide a promising strategy for mitochondria-targeted metal ion fluorescent probes.

## 2. Results and Discussion

### 2.1. Selectivity Toward Metal Ions

**Figure 1:**
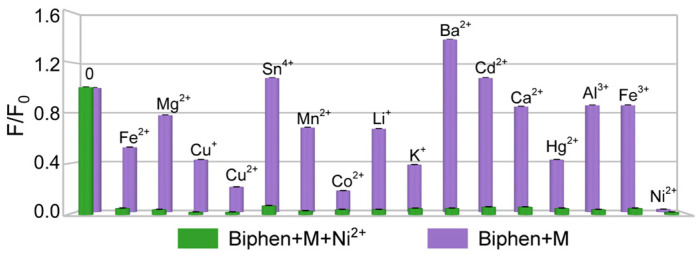
Fluorescence response of Biphen (10 μM) in CH_3_OH–H_2_O (9:1, *v*/*v*) to 10 equiv. of various metal ions (purple bars) and to Ni^2+^ (10 μM) in the presence of 10 equiv. of competing metal ions (green bars). F_0_ and F denote the fluorescent intensities of Biphen in the absence and presence of metal ions, respectively. λ_ex_ = 350 nm. Error bars represent standard deviation (SD), and all experiments were conducted in three replicates.

Prior to cation screening, we explored the effects of common anions including acetate on the fluorescence behavior of Biphen. All examined anions showed almost no interference, which ensured stable detection conditions. Subsequently, the selectivity of Biphen toward various metal cations was evaluated by fluorescence spectroscopy in CH_3_OH–H_2_O (9:1, *v*/*v*). As shown in Figure 1 and Figure S4, 10 equiv. of aqueous Fe^2+^, Mg^2+^, Cu^+^, Cu^2+^, Sn^4+^, Mn^2+^, Co^2+^, Li^+^, K^+^, Ba^2+^, Cd^2+^, Ca^2+^, Hg^2+^, Al^3+^ and Fe^3+^ was added to a methanol solution of Biphen (10 μM), producing negligible changes in fluorescence intensity. In contrast, the fluorescence intensity at 426 nm decreased by a factor of 48 immediately upon the addition of Ni^2+^ in CH_3_OH–H_2_O at room temperature (purple bar). This phenomenon may be attributed to the paramagnetic nature of Ni(II) ions, which can induce strong fluorescence quenching of nearby fluorophores via electron or energy transfer [49]. Furthermore, to evaluate the practical applicability of Biphen for Ni^2+^ detection, competition experiments were performed to examine the influence of other metal ions on the Ni^2+^ sensing response. In these experiments, equal concentrations of aqueous interfering ions and Ni^2+^ were added to a CH_3_OH solution of Biphen (10 μM). The results showed that the fluorescence intensity remained nearly unchanged compared with that observed in the absence of competing ions in the Ni^2+^ solution (green bar). For comparison, the fluorescence response of Phen (10 µM) toward 1.0 equiv. of various metal ions was also investigated in CH_3_OH–H_2_O (9:1, *v*/*v*). However, Phen exhibited no selectivity toward these metal cations (Figure S5). All fluorescence measurements for metal ion detection were performed in at least three independent replicates to ensure reproducibility and reliability.

Given the strong coordination ability of phenanthroline toward metal ions [15], we proposed that Biphen interacts with Ni^2+^ to form a coordination complex. To further verify this hypothesis, *N*,*N*,*N*′,*N*′-tetrakis(2-pyridylmethyl) ethylenediamine (TPEN) was introduced into the Biphen/Ni^2+^ methanol–water solution. Upon addition of TPEN, the fluorescent emission was restored (Figure S6A), indicating that Biphen exhibits reversible sensing behavior and a promising recyclable detection capability. Based on the above findings, the designed probe exhibits significant potential for the selective detection of Ni^2+,^ suggesting promising applicability in biological and environmental systems. The probe’s response time to Ni^2+^ was also evaluated (Figure S6B). The fluorescence intensity reached a minimum within 15 s and remained stable thereafter, indicating the rapid response of Biphen to Ni^2+^. Interestingly, the fluorescence intensity increased upon the addition of the chelating agent (TPEN) to the Biphen/Ni^2+^system. However, it did not fully recover to the initial level, indicating that TPEN exhibits a stronger chelating affinity for Ni^2+^ than Biphen [29]. Subsequently, upon reintroduction of Ni^2+^ ions, the fluorescence intensity decreased to its minimum value again, demonstrating that Biphen exhibits good reversibility and recyclability for Ni^2+^ detection.

**Figure 2:**
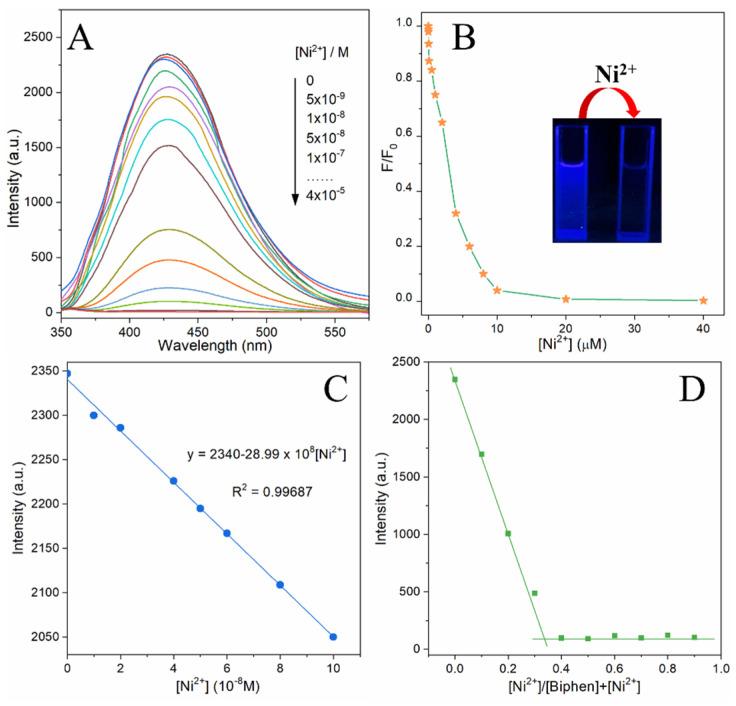
(**A**) Fluorescence emission spectra of Biphen (10 μM) in CH_3_OH–H_2_O (9:1, *v*/*v*) with increasing concentrations of Ni^2+^; λ_ex_ = 350 nm. (**B**) Plot of relative fluorescence intensities (F/F_0_) of Biphen versus Ni^2+^ concentrations. F_0_ and F represent the fluorescence intensities in the absence and presence of Ni^2+^, respectively. Inset: photographs of Biphen and Biphen + Ni^2+^ under UV illumination (365 nm). (**C**) Calibration curve for Ni^2+^ detection by Biphen-Ni^2+^ in CH_3_OH–H_2_O (9:1, *v*/*v*). The detection limit was calculated as DL = K × SD/S = 3 × 4.195/28.99 × 10^8^ = 4.34 × 10^−9^ M. (**D**) Job’s plot for the complexation between Biphen and Ni^2+^. The total concentration of Biphen and Ni^2+^ was 10 μM.

To determine the fluorescent response range of Biphen towards Ni^2+^, the fluorescence emission spectra of Biphen (10 μM) in CH_3_OH–H_2_O (9:1, *v*/*v*) were recorded in the presence of increasing concentrations of Ni^2+^. As shown in Figure 2A, the fluorescence intensity of Biphen gradually decreased with increasing Ni^2+^ concentration, and pronounced fluorescence quenching was observed at 6 μM. Notably, the fluorescence change was visible to the naked eye (Figure 2B, images), suggesting that the probe is potentially suitable for instrument-free Ni^2+^ detection. The detection limit (DL) of Ni^2+^ using Biphen was subsequently determined according to the following equation [29]: DL = K × SD/S (K = 3), where SD represents the standard deviation of the blank solution, and S is the slope of the calibration curve (Figure 2C).

The DL of the probe for Ni^2+^ was determined to be 4.34 × 10^−9^ M (*R*^2^ = 0.9969, equivalent to 0.25 μg/L), indicating excellent sensitivity and suitability for ultratrace detection of Ni^2+^. This value is well below the permissible limits for nickel in drinking water, including those set by the World Health Organization (WHO, 20 ng/L, SDWA) and the Chinese Environmental Quality Standard (0.5 mg/L of total Ni content, GB 25467-2010) [50]. To further investigate the binding mechanism, Job’s plot analysis was performed (Figure 2D). The fluorescence intensity at 426 nm was plotted against the molar fraction of Ni^2+^ while maintaining a constant total concentration (1 × 10^−5^ M). An inflection point was observed at a molar fraction of 0.34, indicating a 2:1 binding stoichiometry between Biphen and Ni^2+^. Accordingly, a possible binding mode of Biphen with Ni^2+^ is illustrated in Scheme S1. This assignment was further supported by high-resolution mass spectrometry (HRMS) (Figure S7), which showed a peak at *m*/*z* = 809.1496 corresponding to [2Biphen + Ni + Cl]^+^). For the fluorescence quenching system with a binding ratio of 2:1 between probe and metal ion, the binding constant was calculated using the modified Benesi–Hildebrand equation. We plotted 1/(F_0_ − F) against 1/[Ni^2+^]^2^, and the stable binding constant was determined from the intercept and slope of the fitted linear curve. Benesi–Hildebrand analysis of the titration profile based on 2:1 binding mode resulted in a nice linearity, further demonstrating the 2:1 binding stoichiometry of Biphen and Ni^2+^, and the binding constant was estimated to be 1.39 × 10^11^ M^−2^, with a correlation coefficient of R = 0.99755 (Figure S8). The higher binding constants indicate that the stability of the complexes formed by the Biphen probe with Ni^2+^ is higher [51].

Subsequently, the performance of the Biphen probe was compared with that of previously reported Ni^2+^ fluorescent probes, as summarized in Table 1. The results indicate that Biphen offers clear advantages in terms of detection limit and linear response range. Most reported probes are limited by relatively high detection limits (typically in the μM or high nM range), lack of reversibility, or insufficient selectivity, often exhibiting cross-reactivity with competing metal ions [21,25,30]. Although a few systems achieve nM-level sensitivity [23,31,37], integrating high selectivity, ultratrace sensitivity, and reversible response remains uncommon. In contrast, Biphen exhibits an ultralow detection limit of 4.34 × 10^−9^ M, along with excellent reversibility and high selectivity toward Ni^2+^, distinguishing it from most reported systems. These attributes highlight its strong potential for practical applications, including environmental monitoring, trace Ni^2+^ analysis in food and biological samples, and reversible subcellular imaging in living cells, thereby enabling real-time, repeatable detection.

### 2.2. Practical Applications in Biological Systems

**Figure 3:**
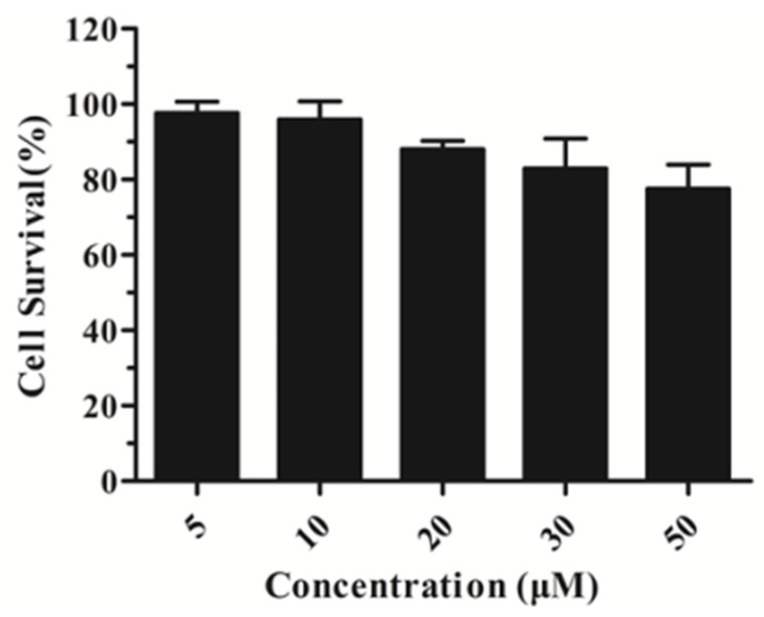
Cell viability of HepG-2 cells treated with different concentrations of Biphen for 24 h, determined by the MTT assay. Error bars represent standard deviation (SD), and all experiments were conducted in four replicates.

Considering the essential role of mitochondria in cellular function and their involvement in nickel-induced toxicity, a series of experiments was conducted to evaluate the practical applicability of Biphen for Ni^2+^ detection in mitochondria. Because an ideal cellular probe should exert minimal perturbation to living systems at working concentrations, we first evaluated the cytotoxicity of Biphen using the standard (4,5-dimethyl-2-thiazolyl)-2,5-diphenyl-2-H-tetrazolium bromide (MTT) assay in HepG-2 cells [52]. The time-dependent effect of Biphen on cell viability at 37 °C is shown in Figure 3. Notably, at a staining concentration of 10 μM, no significant cytotoxicity was observed, with cell viability remaining ≥95% after 24 h of incubation. These results indicate that Biphen exhibits low cytotoxicity and is suitable for fluorescence imaging in living cells under the applied conditions.

**Figure 4:**
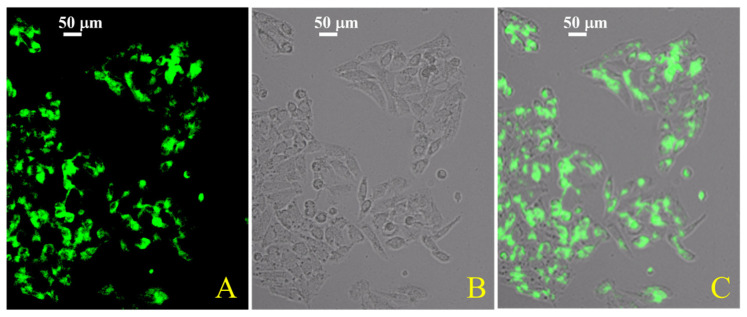
Fluorescence images of HepG-2 cells stained with Biphen (10 μM) for 15 min, obtained using an inverted fluorescence microscope. (**A**) Dark-field image; (**B**) Bright-field image; (**C**) Merged image of (**A**,**B**).

Following this, the cell staining capability of Biphen was examined using an inverted fluorescence microscope (Leica DMi8, Wetzlar, Germany). Under selective excitation at 426 nm, HepG-2 cells incubated with 10 μM Biphen for 15 min at 37 °C exhibited clear intracellular fluorescence signals (Figure 4). Brightfield imaging confirmed that the cells remained viable throughout the imaging experiments. Preliminary observations further indicated that the fluorescence signals were primarily localized in mitochondrial regions (Figure 4).

**Figure 5:**
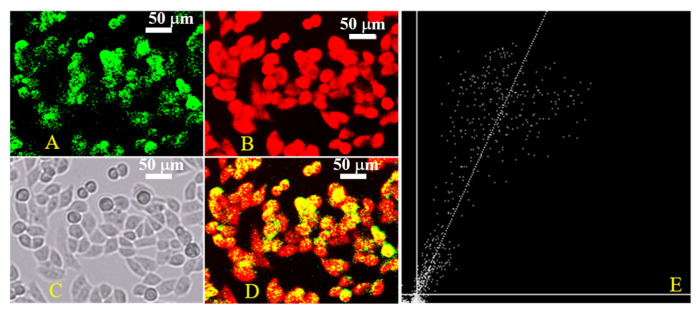
Confocal fluorescence images of HepG-2 cells stained with Biphen (10 μM). (**A**) Biphen fluorescence channel; (**B**) MitoTracker Red CMXRos channel; (**C**) bright-field image; (**D**) merged image of (**A**,**B**); (**E**) fluorescence intensity profile demonstrating the co-localization of Biphen with MitoTracker Red CMXRos.

To determine the precise intracellular localization of Biphen, co-localization imaging experiments were performed using MitoTracker Red CMXRos, a commercially available mitochondrial marker (λ_ex_ = 579 nm, λ_em_ = 599 nm). HepG-2 cells were first incubated with Biphen (10 μM) in PBS (Phosphate-Buffered Saline, pH = 7.4). After incubation, the cells were washed 3 times with PBS to remove excess probe, then incubated with Mito Tracker Red CMXRos (10 μM) for 5 min. As shown in Figure 5, a strong overlap between the fluorescence signals of Biphen and Mito-Tracker Red CMXRos was observed, indicating that Biphen is selectively localized in mitochondria. Pearson’s colocalization coefficient, which quantifies the correlation between the intensity distributions of the Biphen and MitoTracker signals, was calculated to be 0.8312, confirming a high degree of mitochondrial colocalization.

**Figure 6:**
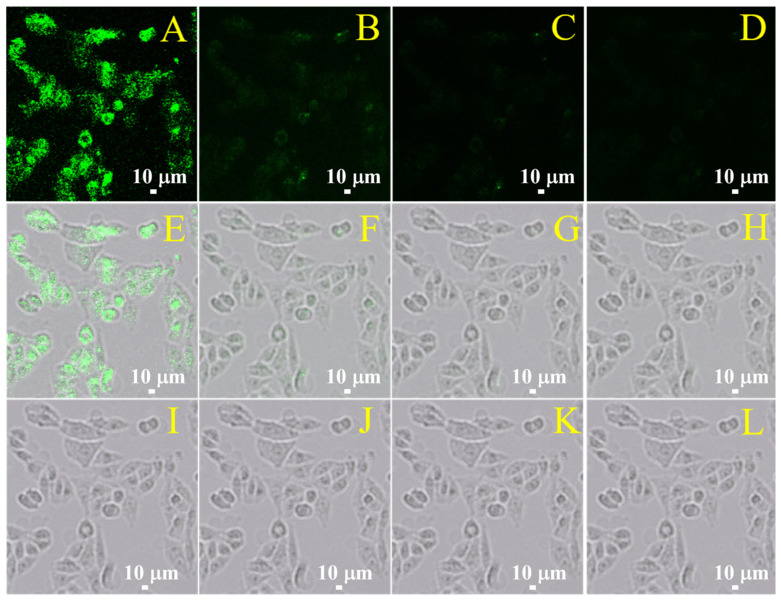
Inverted fluorescence microscopy images of HepG-2 cells stained with Biphen in the presence of increasing concentrations of Ni^2+^: (**A**–**D**) Dark-field fluorescence images; (**E**–**H**) merged images; (**I**–**L**) bright-field images. Ni^2+^ concentrations for panels (**A**–**D**) are 0 mol/L, 5 × 10^−9^ mol/L, 1 × 10^−8^ mol/L, 5 × 10^−8^ mol/L, respectively.

**Figure 7:**
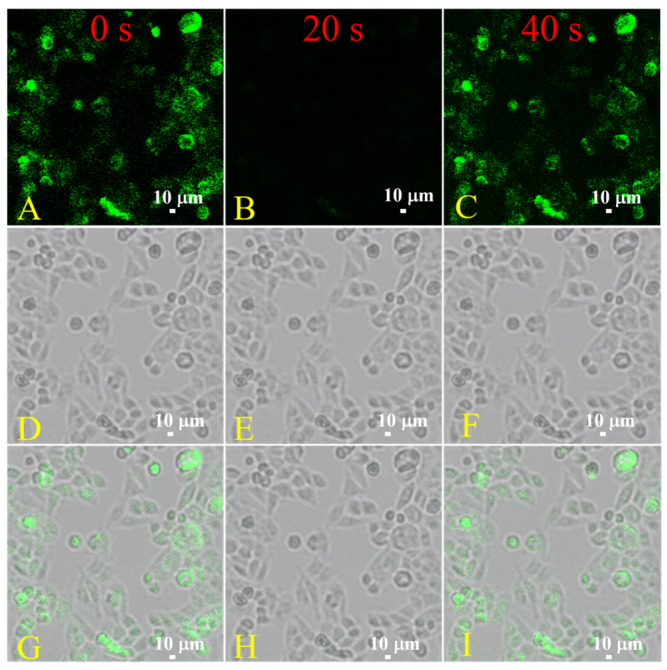
Inverted fluorescence microscopy images showing reversible detection of Ni^2+^ in mitochondria of living HepG-2 cells using Biphen (10 μM). (**A**–**C**) Dark-field fluorescence images; (**D**–**F**) bright-field images; (**G**–**I**) merged images of the fluorescence and bright-field channels. The concentration of Ni^2+^ and TPEN was both 5 μM.

For fluorescent probes used for living-cell imaging, photostability is a critical parameter. Therefore, the photostability of Biphen was evaluated by continuous laser scanning of stained cells. Encouragingly, the results showed that Biphen exhibits excellent photostability under repeated laser irradiation (Figure S9). The combined features of high fluorescence brightness, specific mitochondrial localization, and remarkable photostability prompted us to investigate its utility for detecting Ni^2+^ in mitochondria. Given the low detection limit of Biphen for Ni^2+^ (4.34 × 10^−9^ M), a concentration gradient experiment was performed (Figure 6), demonstrating that the probe can detect Ni^2+^ in the mitochondria of living cells. Based on the kinetic and reversibility studies described above, we hypothesized that Biphen could function as a reversible probe for Ni^2+^ detection in mitochondria. To verify this hypothesis [53], time-dependent confocal fluorescence images were acquired after cells were incubated with Biphen under the same experimental conditions (Figure 7). Upon addition of 5 μM Ni^2+^ in PBS (pH = 7.4), a rapid and pronounced quenching of the intracellular fluorescent was observed within 0–20 s. Subsequently, after introducing 5 μM TPEN in PBS (pH = 7.4), green fluorescence rapidly reappeared in the mitochondrial region within 40 s. These results demonstrate the reversible on-off-on fluorescence response of Biphen toward Ni^2+^ in mitochondria, highlighting its potential for biological sensing and diagnostic applications.

## 3. Materials and Methods

### 3.1. Chemicals and Instruments

All the reagents were obtained from commercial sources and used without further purification. The organic solvents were of analytical-grade quality. In general, the final compounds were purified by column chromatography on silica gel (200–300 mesh). Reactions were monitored by using thin-layer chromatography (TLC) (Qingdao Haiyang silica gel reagent factory GF254, Qingdao, China).

^1^H-NMR and ^13^C-NMR spectra were obtained on Bruker-400 (Billerica, MA, USA) and referenced to 7.27 ppm for chloroform solvent with tetramethylsilane (TMS) as an internal standard (0 ppm). Chemical shifts were reported in parts per million (ppm, δ) downfield from TMS. Proton coupling patterns are described as singlet (s), doublet (d), triplet (t), multiplet (m). Melting points were measured on an Electrothemal SGW-X4 microscopy digital melting point apparatus (Staffordshire, UK) and are uncorrected. ESI/HRMS spectra were obtained on a micrOTOF-Q II mass spectrometer (BrukerDaltonik, Bremen, Germany). The fluorescence spectra were obtained using a Hitachi F-4500 fluorescence spectrophotometer (Tokyo, Japan) with a quartz cuvette (path length =1 cm). For all measurements, both excitation and emission slit widths were 10 nm.

### 3.2. Synthesis

Synthesis of 2,3′-bi(1,10-phenanthroline) (Biphen): Under a N_2_ atmosphere, 1,10-phenanthroline (0.2 mmol), Isopropanol (0.6 mmol), NaOH (0.2 mmol), Pd(OAc)_2_ (3 mol %), and 1,4-dioxane (1.0 mL) were added into a Schlenk tube (50 mL), successively. Then, the Schlenk tube was closed and the resulting mixture was stirred at 125 °C for 16 h. After cooling down to room temperature, the reaction mixture was concentrated by removing the solvent under vacuum. Finally, the residue was purified by preparative TLC on silica to give the pure product as a brown solid (yield 32%): m.p. 207–208 °C; ^1^H NMR (400 MHz, CDCl_3_): 9.87 (s, 1H), 9.46 (s, 1H), 9.26 (d, *J* = 2.9 Hz, 1H), 9.20 (d, *J* = 3.1 Hz, 1H), 8.39 (d, *J* = 8.3 Hz, 1H), 8.31 (d, *J* = 8.4 Hz, 1H), 8.26 (t, *J* = 6.7 Hz, 2H), 8.02 (d, *J* = 8.8 Hz, 1H), 7.86–7.75 (m, 3H), 7.68–7.60 (m, 2H). ^13^C NMR (101 MHz, CDCl_3_): δ 154.53, 150.45, 150.33, 148.90, 146.45, 146.27, 146.15, 146.06, 137.32, 136.30, 136.05, 136.04, 135.78, 134.26, 129.20, 129.10, 128.69, 128.06, 127.33, 126.93, 126.36, 123.21, 120.84. HRMS (ESI): Calcd. for 359.1291 [M+H]^+^; found: *m*/*z* 359.1289. The relevant spectra are available in the Supporting Information (Figures S1–S3).

### 3.3. Procedures of the Metal Ion Sensing

Stock solutions (100 μM) of Cu^2+^, Hg^2+^, Mg^2+^, Ca^2+^, Cd^2+^, Sn^4+^, Fe^3+^, Al^3+^, Fe^2+^, Mn^2+^, Co^2+^, Cu^+^, Li^+^, K^+^, Ni^2+^, and Ba^2+^ were prepared from their chloride salts. The stock solution of Biphen was prepared at 100 μM in CH_3_OH. The test solution of Biphen (10 μM) in 2 mL of aqueous methanol solution was prepared by placing 0.2 mL of Biphen stock solution and 0.2 mL of the aqueous sample solution in 1.6 mL CH_3_OH. The mixture solution was shaken well and kept at room temperature for 10 min before the spectra were recorded.

### 3.4. Cell Staining and Viability Assays

HepG-2 cells were cultured in Dulbecco’s modified Eagle’s medium (DMEM) supplemented with 10% fetal bovine serum (FBS, GIBCO, Waltham, MA, USA), penicillin and streptomycin. The cells were seeded in a 24-well glass-bottom plate at a density of 10,000 cells/well and incubated at 37 °C for 24 h with 5% CO_2_ and 95% air in order to allow ~90% cell confluence. Probe Biphen was added at a final concentration of 10 μM to the culture and incubated for 5 min under the same conditions as above. The cells were imaged with a Leica DMi8 microscope (Wetzlar, Germany). For the staining experiment, the cells were incubated with Red CMXRos (Servicebio, Dongguan, China), which is an infrared dye (λ_ex_ = 579 nm, λ_em_ = 599 nm), in DMEM at 37 °C for 30 min with 5% CO_2_ and 95% air.

For the measurement of the toxicity of the Biphen probe, HepG-2 cells were first grown to the logarithmic growth phase and seeded into 96-well plates at a density of 1000 cells/well and incubated at 37 °C for 24 h with 5% CO_2_ and 95% air. The Biphen probe was added with different indicated concentrations and incubated for another 12, 24 or 48 h. Cell viability was determined by using the methylthiazolyldiphenyl-tetrazolium bromide (MTT) assay as previously described. Each individual cytotoxic experiment was repeated four times.

### 3.5. Microscopy and Micrograph Analyses

HepG-2 cells were luminescently imaged on a Leica inverted fluorescence microscope with 10× lenses. Quantification of the micrograph fluorescence intensity was performed via an ImageJ (Java 1.6.0_20) plug-in ROI (region of interesting) manager. At least 3 micrographs for each experiment were required, and 10 data points from each micrograph were obtained.

## 4. Conclusions

In summary, 2,3′-bi(1,10-phenanthroline) (Biphen) was synthesized via a hydrogen transfer-mediated coupling strategy in a single step. For the first time, this compound was developed as a fluorescence probe for Ni^2+^, exhibiting high sensitivity and a pronounced fluorescence-quenching response toward Ni^2+^ relative to other metal ions. The probe shows a detection limit as low as 4.34 × 10^−9^ M in CH_3_OH–H_2_O solution. In addition, the probe enables ultratrace detection of Ni^2+^ in mitochondria through a reversible on-off-on fluorescence switching mechanism and exhibits excellent photostability. Owing to these properties, Biphen shows significant potential for Ni^2+^ recognition and cancer cell imaging, providing a useful platform for mitochondrial studies and diagnosis. Furthermore, this probe offers a promising strategy for selective, ultratrace detection of Ni^2+^ in aqueous and biological systems. Despite its favorable sensing performance, a key limitation remains: optimal detection is achieved in a CH_3_OH–H_2_O (9:1, *v*/*v*) mixed solvent system, limiting its direct application in fully aqueous environments. Future work will focus on rational structural modification of Biphen to enhance its aqueous solubility, thereby broadening its applicability in environmental monitoring and more complex biological fluid systems.

## Figures and Tables

**Table 1:** Comparison of reported fluorescent probes for Ni^2+^ detection.

Probe System	Detection Medium	Selectivity	LOD (M)	Reversibility	Ref.
Quinoxaline-based	CH_3_CN–H_2_O	Ni^2+^	1.47 × 10^−6^	No	[4]
Poly(arylene ether)-terpyridine	DMF	Ni^2+^	1 × 10^−8^	No	[10]
Pyrimidine-based	DMSO–H_2_O	Ni^2+^	0.233 × 10^−6^	No	[19]
Isoniazid-based Schiff base	EtOH–H_2_O	Cu^2+^, Ni^2+^, Hg^2+^	7.685 × 10^−7^	Yes	[21]
Schiff base probe	H_2_O	Ni^2+^, CN^−^	57 × 10^−9^	No	[22]
Rhodamine-based	CH_3_CN–H_2_O	Ni^2+^	26.2 × 10^−9^	No	[23]
Benzimidazole-based	H_2_O	Ni^2+^	0.14 × 10^−6^	No	[24]
CdS quantum dots	H_2_O	Co^2+^, Ni^2+^	5 × 10^−8^	Yes	[25]
Hydrazine carbothioamide-based	CH_3_CN–H_2_O	Ni^2+^	7.9 × 10^−8^	No	[26]
Boron nitride quantum dots	Phosphate buffer	Ni^2+^	0.1 × 10^−6^	No	[27]
Imidazole-containing amide	H_2_O	Ni^2+^	2.82 × 10^−6^	No	[28]
Pyridine-based	CH_3_CN	Ni^2+^	1.83 × 10^−6^	Yes	[29]
Indole-Schiff base	DMSO:H_2_O	Ni^2+^, Co^2+^, Cd^2+^, Cu^2+^	2.04 × 10^−6^	No	[30]
Quaternary ammonium salt-based	CH_3_CN–H_2_O	Ni^2+^	17 × 10^−9^	No	[31]
Imidazole-modified carbon dots	BR buffer solution	Ni^2+^	0.93 × 10^−3^	No	[32]
Phenanthroline-based	EtOH	Ni^2+^	61 × 10^−9^	No	[37]
Biphen (this work)	CH_3_OH–H_2_O	Ni^2+^	4.34 ×10^−9^	Yes	This work

## Data Availability

The original contributions presented in the study are included in the article and Supplementary Materials. Further inquiries can be directed to the corresponding authors.

## Supplementary Materials

The following supporting information can be downloaded at: https://www.mdpi.com/article/10.3390/molecules31111815/s1, Figure S1: ^1^H NMR spectra of Biphen; Figure S2: ^13^C NMR spectra of Biphen; Figure S3: HRMS spectra of Biphen; Figure S4: The fluorescence intensity of Biphen (10 µM) upon addition of 10 equiv. of various metal ions in CH_3_OH–H_2_O (9:1, *v*/*v*), λ_ex_ = 350 nm; Figure S5: The fluorescence intensity of Phen (10 µM) upon addition of 1.0 equiv. of various metal ions in CH_3_OH–H_2_O (9:1, *v*/*v*). λ_ex_ = 350 nm; Figure S6: (A) Fluorescence spectra of Biphen (10 µM) in the presence of Ni^2+^ (1.0 equiv.) and TPEN (1.0 equiv.) in CH_3_OH–H_2_O (9:1, *v*/*v*). (B) Plots of fluorescence intensity of Biphen (10 μM) vs. the reaction time in the presence of Ni^2+^ (1.0 equiv.) in CH_3_OH–H_2_O; Figure S7: HRMS spectra of Biphen in the presence of NiCl_2_∙6H_2_O (1.0 equiv); Figure S8: The Benesi-Hildebrand equation for the interaction of compound Biphen with Ni^2+^; Figure S9: Normalized intensity of Biphen over continued laser scanning; Scheme S1. Proposed possible binding modes of Biphen with Ni^2+^.
