# Supplementary material for: Phenanthroline-Based Reversible Fluorescent Probe for Ultrasensitive and Selective Detection of Ni2+ and Mitochondrial Imaging
Source: Molecules. 2026 May 25;31(11):1815. doi: 10.3390/molecules31111815 (PMC13258799; doi:10.3390/molecules31111815)
Supplement: Supplementary file 1 [file molecules-31-01815-s001.zip › molecules-4321153-supplementary.pdf]

Supporting Information

Phenanthroline-based reversible Fluorescent Probe for Ultrasensitive and Selective Detection of Ni^{2+} and Mitochondrial Imaging

Jing Huang[1], **Xinyan Yu**[1], **He Zhao**[1,*], **Fenying Kong**[1,*] and **Yong Dai**[1,*]

[1]Chemistry & Chemical Engineering, Yancheng Institute of Technology, Yancheng 224007, China
*Correspondence: zhaohe@ycit.edu.cn (H.Z.); kongfy@ycit.edu.cn (F.K.); dy@ycit.cn (Y.D.)

Table of Contents

Figure S1. ^{1}H NMR spectra of Biphen.
Figure S2. ^{13}C NMR spectra of Biphen.
Figure S3. HRMS spectra of Biphen.
Figure S4. The fluorescence intensity of Biphen (10 μM) upon addition of 10 equiv. of various metal ions in $CH_3OH–H_2O$ (9 : 1, v/v). λ_{ex} = 350 nm.
Figure S5. The fluorescence intensity of Phen (10 μM) upon addition of 1.0 equiv. of various metal ions in $CH_3OH–H_2O$ (9 : 1, v/v). λ_{ex} = 350 nm.
Figure S6. (**A**) Fluorescence spectra of Biphen (10 μM) in the presence of Ni^{2+} (1.0 equiv.) and TPEN (1.0 equiv.) in $CH_3OH–H_2O$ (9 : 1, v/v). (**B**) Plots of fluorescence intensity of Biphen (10 μM) *vs* the reaction time in the presence of Ni^{2+} (1.0 equiv.) in $CH_3OH–H_2O$.
Figure S7. HRMS spectra of Biphen in the presence of $NiCl_2·6H_2O$ (1.0 equiv).
Figure S8. The Benesi-Hildebrand equation for the interaction of compound Biphen with Ni^{2+}.
Figure S9. Normalized intensity of Biphen over continued laser scanning.
Scheme S1. Proposed possible binding modes of Biphen with Ni^{2+}.

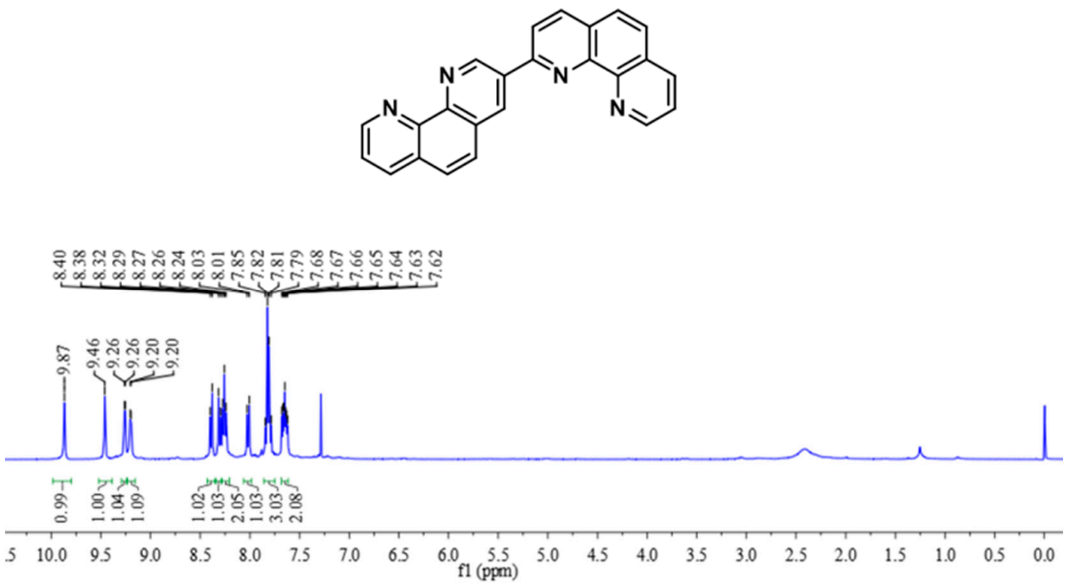

Figure S1. ^1H NMR spectra of Biphen.

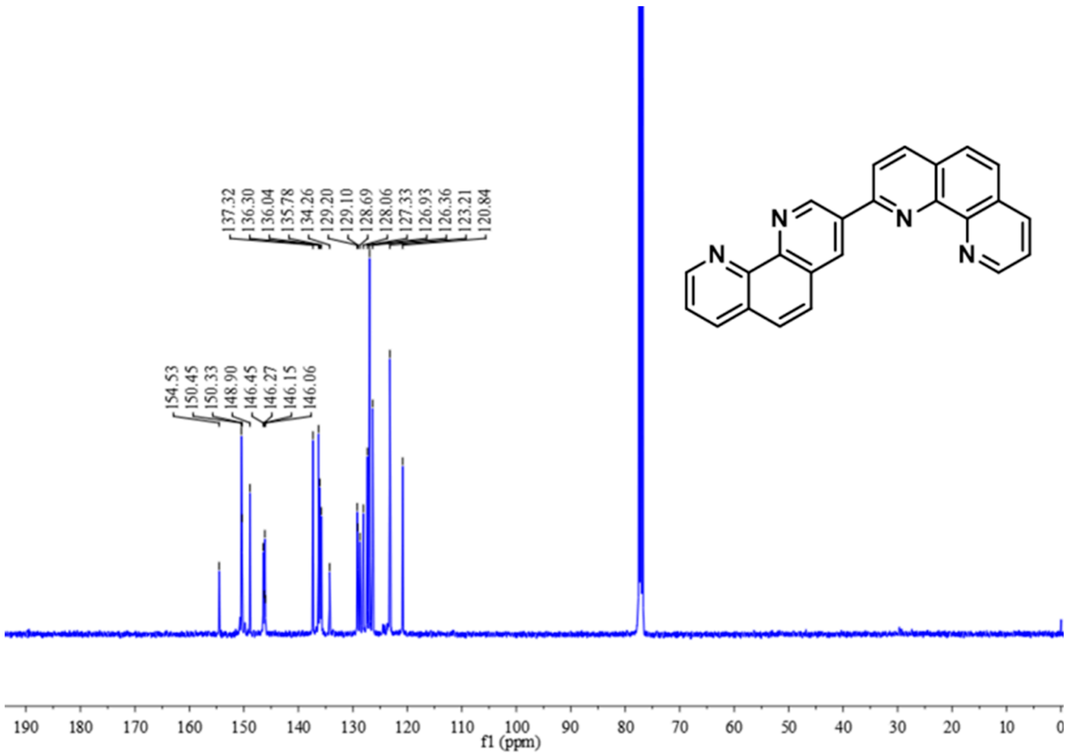

Figure S2. ^{13}C NMR spectra of Biphen.

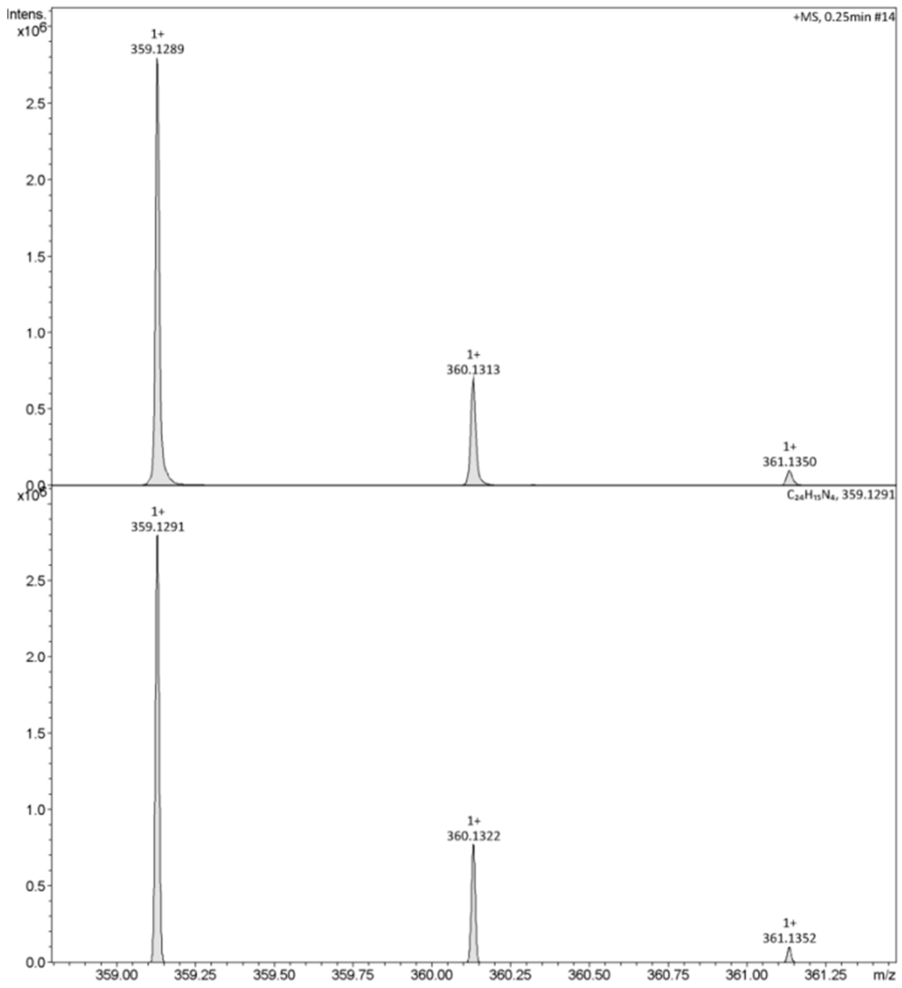

Figure S3. HRMS spectra of Biphen.

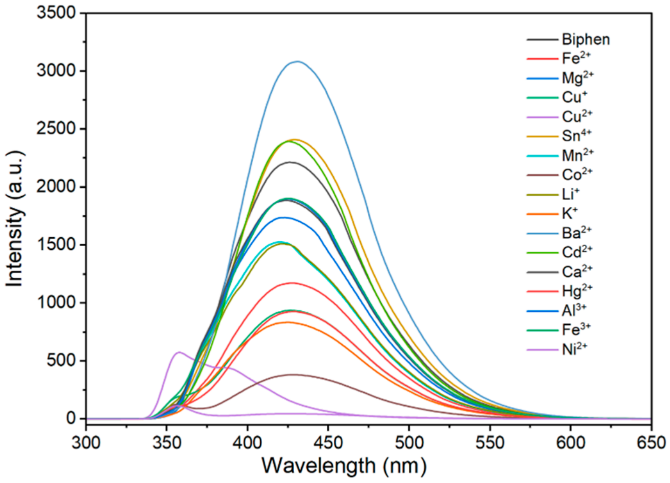

Figure S4. The fluorescence intensity of Biphen (10 μM) upon addition of 10 equiv. of various metal ions in $CH_3OH–H_2O$ (9 : 1, v/v). λ_{ex} = 350 nm.

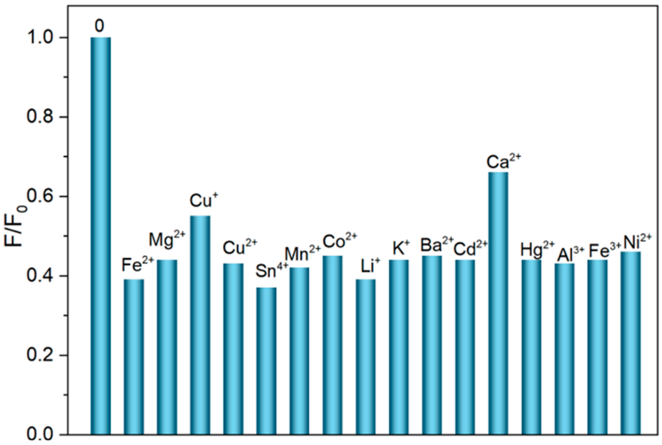

Figure S5. The fluorescence intensity of Phen (10 μM) upon addition of 10 equiv. of various metal ions in $CH_3OH–H_2O$ (9 : 1, v/v). λ_{ex} = 350 nm.

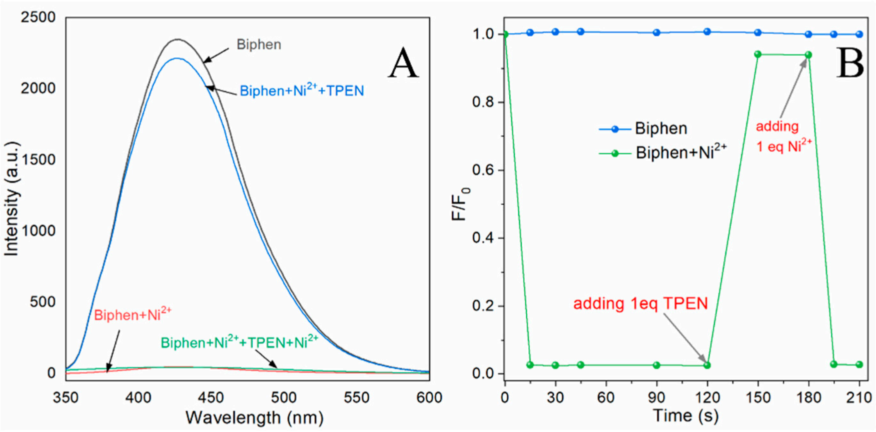

Figure S6. (**A**) Fluorescence spectra of Biphen (10 μM) in the presence of Ni^{2+} (1.0 equiv.) and TPEN (1.0 equiv.) in CH_3OH–H_2O (9 : 1, v/v). (**B**) Plots of fluorescence intensity of Biphen (10 μM) *vs* the reaction time in the presence of Ni^{2+} (1.0 equiv.) in CH_3OH–H_2O.

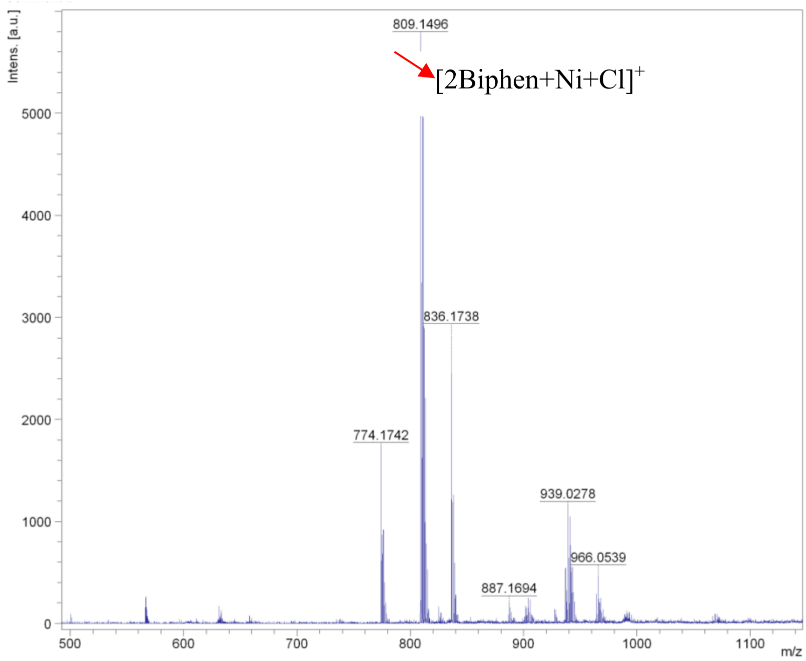

Figure S7. HRMS spectra of Biphen in the presence of $NiCl_2 \cdot 6H_2O$ (1.0 equiv).

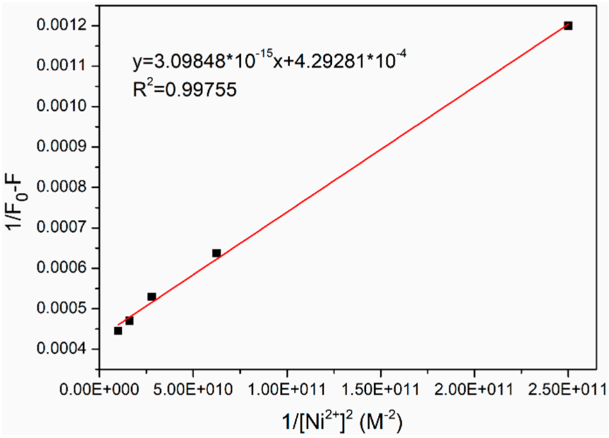

Figure S8. The Benesi-Hildebrand equation for the interaction of compound Biphen with Ni^{2+}.

The binding constants for sensor L-metal complexes have been determined using Benesi-Hildebrand relation. For 2:1 complex the linear Benesi-Hildebrand equation can be written as follows:

$$\frac{1}{F_0 - F} = \frac{1}{F_0 - F_{min}} + \frac{1}{K(F_0 - F_{min})} \times \frac{1}{[\mathrm{M}^{n+}]^2}$$

where F_0 is the fluorescence intensity of the blank sample, F is the fluorescence intensity in the presence of the quencher, F_{min} is the minimum fluorescence intensity in the presence of excess quencher and $[M^{n+}]$ is the concentration of the quencher. By plotting $1/[Ni^{2+}]^2$ on the x-axis against $1/(F_0\text{-}F)$ on the y-axis, a linear fit is obtained. The binding constant (Ka) is then derived from the ratio of the y-intercept to the slope.

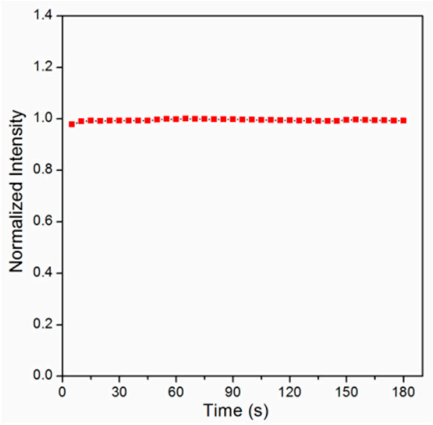

Figure S9. Normalized intensity of Biphen over continued laser scanning.

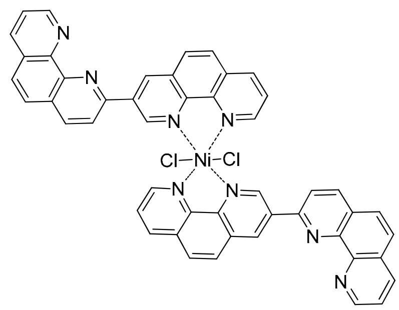

Scheme S1. Proposed possible binding modes of Biphen with Ni^{2+}.
